# Evaluating the quality, feasibility and patient satisfaction of medication history taking by telephone for patients with scheduled admissions: a pilot study

**DOI:** 10.1007/s11096-025-02002-1

**Published:** 2025-09-08

**Authors:** Theresa Terstegen, Janina A. Bittmann, Luise Kauk, Marietta Kirchner, Sebastian Krug, Annika Gauss, Ute Chiriac, Benedict Morath, Walter E. Haefeli, Hanna M. Seidling

**Affiliations:** 1 Heidelberg University, Medical Faculty Heidelberg / Heidelberg University Hospital, Internal Medicine IX – Department of Clinical Pharmacology and Pharmacoepidemiology, Cooperation Unit Clinical Pharmacy, Im Neuenheimer Feld 410, 69120 Heidelberg, Germany; 2 Heidelberg University, Medical Faculty Heidelberg / Heidelberg University Hospital, Institute of Medical Biometry, Im Neuenheimer Feld 103.3, 69120 Heidelberg, Germany; 3 Heidelberg University, Medical Faculty Heidelberg / Heidelberg University Hospital, Internal Medicine IV – Medical Faculty Heidelberg, Department of Gastroenterology, Infectiology and Toxicology, Im Neuenheimer Feld 410, 69120 Heidelberg, Germany; 4 Heidelberg University Hospital, Hospital Pharmacy, Im Neuenheimer Feld 670, 69120 Heidelberg, Germany

**Keywords:** Clinical pharmacy service, Hospitalization, Medication errors, Medication history, Medical history taking, Telepharmacy

## Abstract

**Introduction:**

Medication history taking at hospital admission is still prone to errors. Despite numerous quality improvement initiatives, new strategies to improve medication history taking are still sought and evaluated. Unfortunately, the gold standard research methodology for evaluation is resource-intensive, as it requires each patient to complete two medication history interviews. Therefore, a new study design and quality parameter were developed.

**Aim:**

We aimed to pilot our newly developed study design and quality parameter in a study on medication history taking by telephone.

**Method:**

In this prospective interventional study, patients with scheduled admissions had their medication histories taken either by telephone before admission (intervention) by a pharmacist or in-person by physicians or medical interns upon admission (control). Following the newly developed design, we compared a patients’ new medication histories to the respective pre-visit medication lists available in the medical records to calculate the new endpoint: the difference in the number of updates per patient. Further, we surveyed patients and staff on their satisfaction.

**Results:**

We enrolled 76 intervention and 75 control patients. In the intervention group, a mean of 4.93 (± 4.45, 0–18) updates were found vs. 3.40 (± 3.75, 0–21) in the control group. Accordingly, the incident rate of number of updates per patient was 1.34 times higher in the intervention group (*p* = 0.044). The distribution of the types of updates was similar with the most common type of update being newly initiated medicines in both groups. Medication history taking by telephone took 15.7 ± 9.8 min (mean ± SD), including preparation, interview, and documentation. Survey results showed that intervention patients felt positive about the telephone interviews. Both groups were open to other digital approaches, e.g., online platforms.

**Conclusion:**

The new study design proved feasible to evaluate medication history taking by telephone with comprehensible results. The telephone approach delivered more updates compared to standard care, however, the proposed endpoint needs to be validated against the gold standard before widespread application Patient acceptance for this and other digital approaches was high in both groups.

**Supplementary Information:**

The online version contains supplementary material available at 10.1007/s11096-025-02002-1.

## Impact statements

- We piloted a novel methodology to evaluate medication history taking and proved its practicability.
- Telephone interviews were feasible in our setting and delivered more updated medicines than routine care interviews with the most common type of updates being newly initiated medicines.
- This service was well accepted by patients and attitudes towards telepharmaceutical approaches to obtain medication histories were generally positive.

## Introduction

A medication history on hospital admission is a prerequisite for safe, uninterrupted drug therapy when patients transition from primary to secondary care [1–3]. However, as history taking competes with other clinical procedures and is often interrupted [4, 5], medication errors are common at this stage [6]. In research, the quality of a patient’s medication history is evaluated by taking a *Best Possible Medication History (BPMH)* in addition to the initial history, and comparing the two histories to identify the *number of medication discrepancies* [7–12]. This methodology is laborious, as BPMHs are time-consuming [13, 14]. To overcome this challenge, we developed a new study design that uses pre-existing medication documents as comparators instead of an additional BPMH. Thereby, we derived a new outcome parameter: the *number of updates* between the pre-existing list and the newly acquired medication history [15].

To pilot our new design, we evaluated a telepharmaceutical approach to medication history taking, i. e. conducting the medication history interview by telephone. To our best knowledge, this approach has not yet been investigated in Germany. However, the integration of telepharmacy practices provides a promising avenue for enhancing patient care and allocating resources more efficiently [16–19]. Previous studies on telepharmaceutical approaches to medication history taking resulted in increased completion rates [20] and delivered accurate medication histories while being time-efficient and resource-sparing [21–24]. Particularly telephone medication histories could provide a suitable alternative for patients with planned admissions and mobility problems [25].

### Aim

We aimed to (a) test the feasibility of the new study design and quality parameter, and (b) explore medication history taking by telephone in patients with scheduled admission.

## Method

### Setting and participants

This study was conducted at two gastroenterology wards of a German university hospital between October 2023 and February 2024. Patients ≥ 18 years old and scheduled for inpatient admission were eligible. We excluded patients in whom a medication history could not be taken and patients for whom no available comparator medication history. Multiple admissions during the study period were included only once. The study was registered at the German Clinical Trials Register (#DRKS00032512).

### Study design

**Fig. 1:**
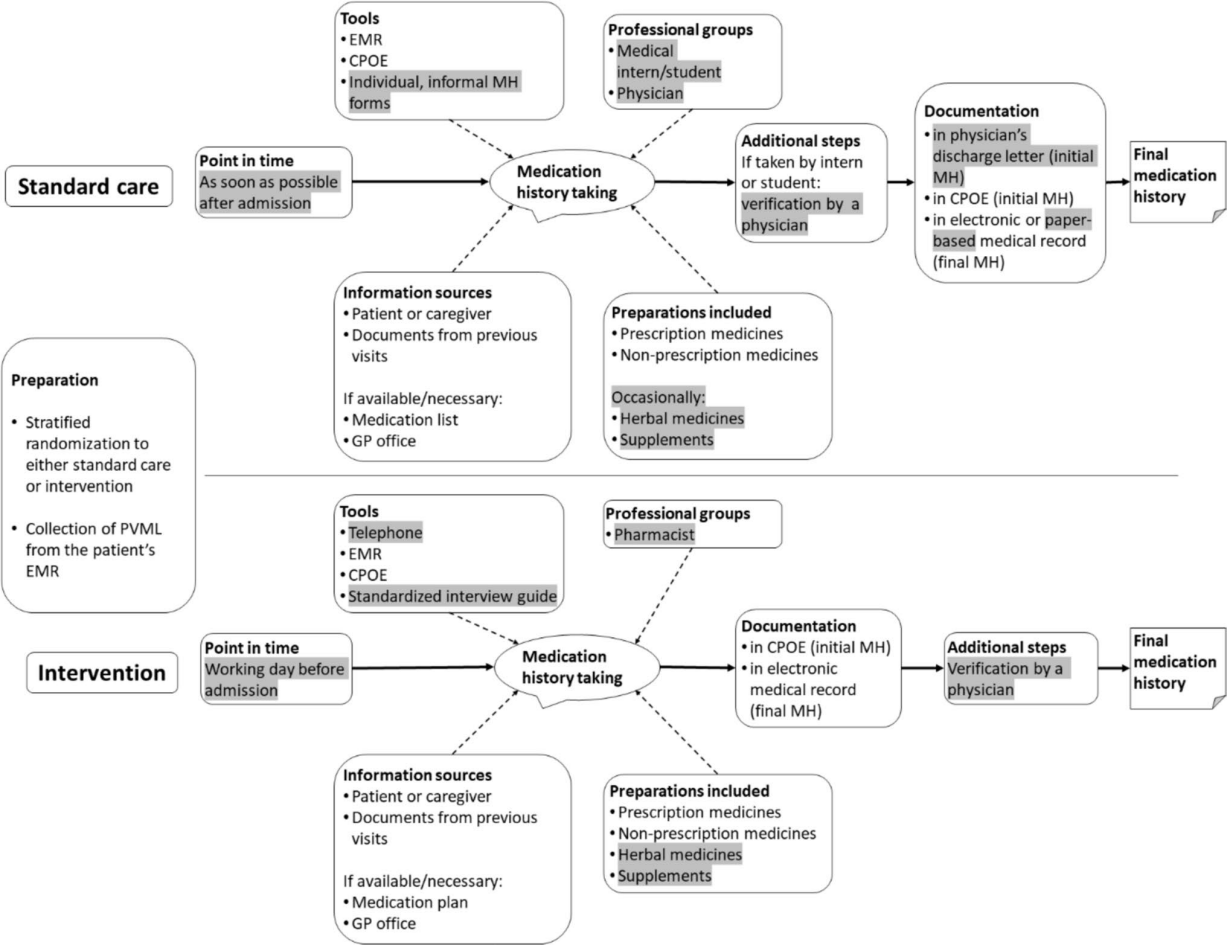
Intervention phase: medication history taking in standard care and intervention group. *Standard-care:* Patients with scheduled admissions usually arrived at the ward in the morning and proceeded to their planned therapeutic or diagnostic procedures. Medication histories were taken by physicians or medical interns or medical students in a face-to-face interview together with the clinical history. If there were waiting times, the medication histories were taken before the procedures. However, in most cases, medication histories were taken and documented in the afternoon, when patients returned to the ward from their procedures. *Telephone intervention:* All patients routinely received a call from the central patient management on the day before their admission to confirm their appointment. During this call, they were informed of an upcoming second call by a pharmacist to take their medication history. In preparation for this interview, the primary investigator (pharmacist) collected the medication information already available in the electronic medical record for each patient in the intervention group. The medication history was collected using a standardized interview guide. All information was documented in the pre-admission medication section of the patient’s electronic medical record. Differences between the two workflows are highlighted in grey. CPOE = computerized physician order entry, EMR = electronic medical record, GP = general practitioner, MH = medication history

The study design has been published previously, this description partly reproduces the wording [15]. In the preparatory phase, the routine process was observed at the study site to identify the pre-visit medication list (PVML), a comprehensive medication document of consistent scope and origin that is generally available for patients at the specific wards. It served as the comparator history for each patient to calculate the primary endpoint. In our setting, the PVML was the most recent in-house discharge letter. In the baseline phase, a pharmacist gathered BPMHs of six patients and compared them to the respective medication histories acquired by standard-care to determine the quality of the current process. During the interventional phase, patients were assigned to the intervention (telephone interview before admission) or control group (standard-care: in-person interview upon admission) (Fig. 1). A stratified randomization was conducted to ensure an equal distribution regarding age, sex, the number of home medicines based on the PVML, and the days elapsed since its documentation. Additionally, a patient survey was conducted using standardized questionnaires for the intervention and control group. Questionnaires consisted of close-ended questions, which were piloted and refined in a previous project [26, 27]. After their medication history had been taken, intervention patients were surveyed on their satisfaction with the telephone interviews regarding timing, location and mode of communication. Control patients were surveyed on-site after admission to a study ward. The questions concerning the telephone interviews were modified for the control group to assess their openness to this approach. Additionally, we briefly assessed patients’ readiness for other digital approaches. All participants provided written, informed consent.

We surveyed staff members (physicians, medical interns) two weeks after the start of the interventional phase and three weeks after its end in a de-briefing meeting. They provided written, informed consent. The questionnaire was developed analogously to the patient survey and assessed the satisfaction with the intervention regarding quality, time savings and potential improvements.

### Endpoints

Our primary endpoint was the *number of updates per patient* between the PVML and the medication history. Every difference between these two lists was considered an *update* and assumed to be an intended therapeutic change by primary care physicians. Therefore, updates are considered corrections of the existing list. Accordingly, a high number of updates reflects better quality. In this way, our endpoint differs from the widely used parameter *medication discrepancies;* where a high number indicates poor quality. Updates in our study included: 1. discontinued medicines, 2. newly initiated medicines, 3. changed medicines. The latter included changes in dose, frequency, time of intake, non-interchangeable dosage form (= formulations that cannot be substituted without potentially altering clinical outcomes), paused/resumed medicines, single-agent products to fixed-dose combinations (and vice versa), and other (e. g., planned discontinuation). Secondary endpoints included the evaluation of updates by categories. Feasibility, patient acceptance and staff satisfaction were explored as tertiary endpoints.

### Data collection

Demographic data, medication data, and the PVML were extracted from the electronic medical record (EMR) and managed in Microsoft® Excel® 2019. The patient’s newly recorded medication history was compared to the PVML to determine the primary endpoint. The time spent on preparation, interview, and documentation was recorded by the pharmacist for the intervention group. The time needed for documentation in the control group was extracted retrospectively from electronic time stamps in the EMR as a proxy.

### Sample size

A power analysis was conducted to determine the sample size using a non-parametric Mann–Whitney-U test at a two-sided significance level of 5%. The analysis showed that a standardized effect of d = 0.5 can be detected with a power of 1-β = 0.826 and a sample size of N = 75 per group. The analysis was conducted in PASS v16.0.12, NCSS, LLC. (Kaysville, UT, USA) using 10,000 simulated data sets.

### Statistical analysis

For the baseline phase, summary statistics were provided for the number of updates. The purpose of this phase was to explore the validity of the primary endpoint by linking it to the gold standard (medication discrepancies); it does not constitute a validation.

For the intervention phase, comprehensive summary statistics were provided for baseline characteristics (sex, age, number of home medicines based on the PVML, days elapsed since the documentation of the PVML) and the endpoints stratified by *group*.

The primary endpoint was compared between the *groups* (intervention vs. control). Since data was not normally distributed (visual inspection), we applied a negative binomial model adjusted for pre-specified confounders (sex, age, days elapsed since documentation, number of home medicines). Further analyses comprised the evaluation of factors impacting the primary endpoint and exploratory subgroup analyses using the same model, but including interaction effects of *group* x *subgroup* factor and their respective *subsets*: sex (male/female), age (continuously), days elapsed since documentation of the PVML (≤ 30, 31–90, ≥ 91), number of home medicines (0–4, 5–9, ≥ 10). The secondary endpoint “ ≥ 1 update” was evaluated in a logistic regression model including *group* as factor and the pre-specified confounders. All secondary endpoints were analysed exploratively by descriptive statistics. The categories “discontinued medicines” and “changed medicines” were only analysed in patients with ≥ 1 medicine documented in the PVML, as otherwise they cannot occur. Tertiary endpoints were described by frequencies and differences explored using Pearson-χ2-tests. A significance level of α = 0.05 was applied. Analyses were conducted in IBM SPSS Statistics Version 28.0 (Armonk, NY, USA).

### Ethics approval

This study was performed in line with the principles of the Declaration of Helsinki. Approval was granted by the Ethics Committee of the Medical Faculty of Heidelberg University (S-625/2022).

## Results

### Patient demographics

In total, 151 were included in the analysis (76 = intervention, 75 = control). The reasons for exclusion mainly related to obtaining consent (Supplement A). The demographic data and the distribution of drug groups according to the anatomical, therapeutic, chemical classification system (ATC) were similar in both groups (Table 1).

**Table 1:** Patient demographics

Variable	Intervention (N = 76)		Control (N = 75)		Overall (N = 151)	
	Mean ± SD or Median [IQR] or N (%)	Range	Mean ± SD or Median [IQR] or N (%)	Range	Mean ± SD or Median [IQR] or N (%)	Range
Age [years]	59.6 ± 14	21–83	57.5 ± 14	19–81	58.6 ± 14	19–83
Sex, female	26 (34.2)	–	26 (34.7)	–	52 (34.4)	–
Days elapsed since documentation of the PVML	64 [110]	6–4158	72 [215]	3–5574	70 [134]	3–5574
Number of home medicines and medical devices based on PVML	6.3 ± 4	0–16	6.3 ± 5	0–20	6.3 ± 4	0–20
Length of stay [days]	1.8 ± 2	0–8	2.2 ± 2	0–12	2.0 ± 2	0–12
Patients responsible for their own medicines [N]	62/67 (92.5)	–	66/75 (88.0)	–	128/142 (90.1)	–
Using aids for organizing medicines	42/75 (56.0)	–	31/68 (45.6)	–	73/143 (51.0)	–
*Percentage of the 5 most common drug groups (ATC) of all medicines recorded [%]*						
A: Alimentary tract and metabolism	36.9	–	34.5	–	35.7	–
C: Cardiovascular system	22.9	–	27.6	–	25.2	–
B: Blood and blood forming organs	11.1	–	9.0	–	10.1	–
N: Nervous system	9.5	–	8.0	–	8.7	–
L: Antineoplastic and immunomodulating agents	5.6	–	4.9	–	5.2	–

ATC = anatomical, therapeutic, chemical classification, IQR = interquartile range, N = number, PVML = pre-visit medication list, SD = standard deviation

### Quality of the medication history

In the baseline phase, we recorded the medication histories of six patients acquired by standard-care and conducted BPMHs on the same patients. Compared to the respective PVMLs, we found a mean of 2.8 (± 2.1, 1–6) updates in standard-care medication histories and 7.0 (± 3.5, 4–13) updates in BPMHs. In total, 33 medication discrepancies were identified between the BPMH and the standard-care histories with a mean number of 6.2 (± 23.1, 4–11). Of these, 22 (66%) could be attributed to medicines not being updated, while 11 (33%) were actual errors. They included missing or incorrect doses or frequencies and medicines erroneously removed from the medication list, despite still being used by the patient.

### Primary endpoint

**Fig. 2:**
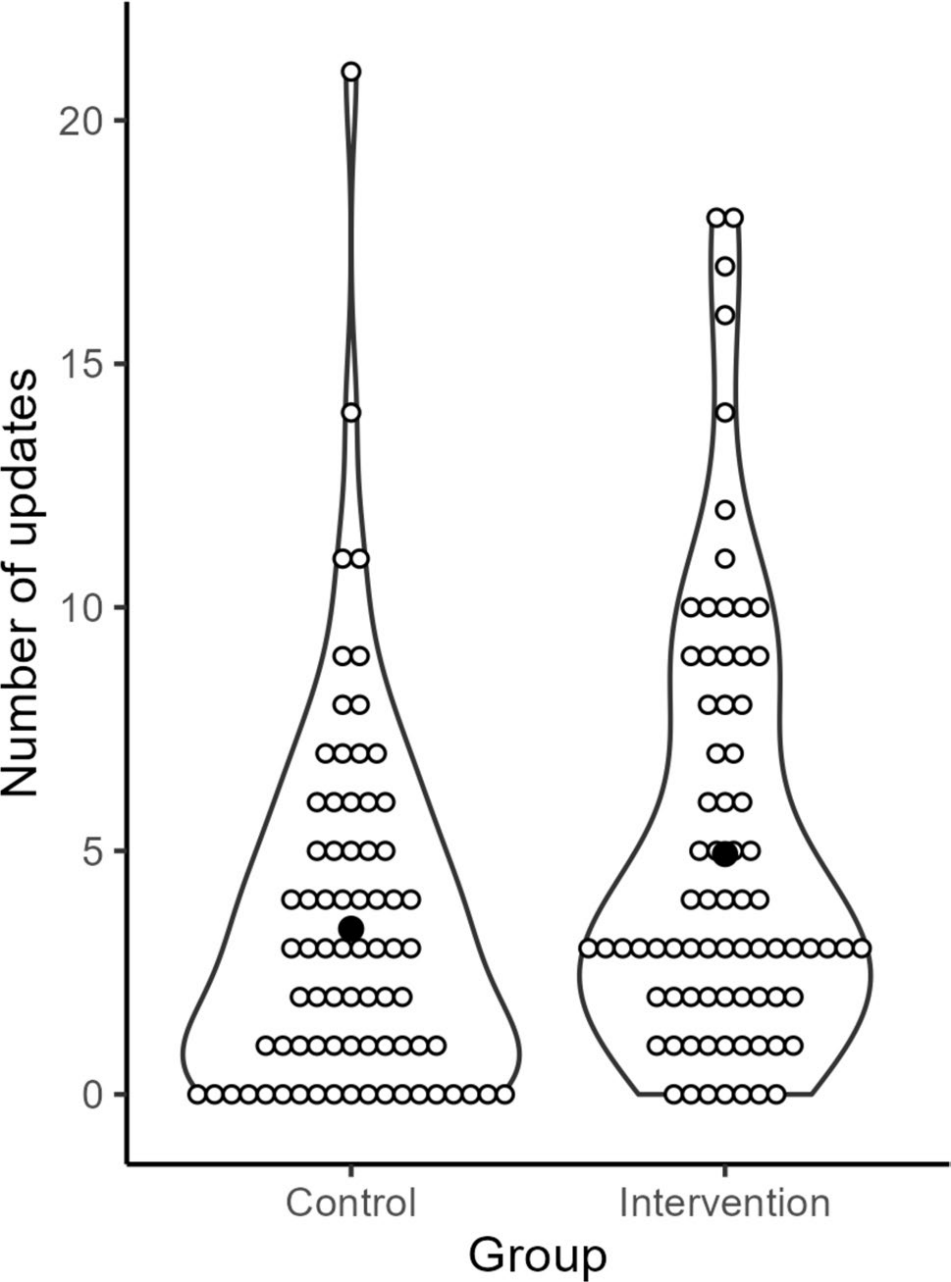
Violin plot of the number of updates in intervention and control group. Filled circle = mean number of updates in the group, empty circle = observed number of updates of the individual participants in the group. Updates were defined as differences between the pre-visit medication list and the newly acquired medication history. They included discontinued medicines, newly initiated medicines, and changed medicines. Figure created with R version 4.1.3 (R Foundation for Statistical Computing, Vienna, Austria)

We found a mean of 4.93 (± 4.45, 0–18) updates in the intervention group vs. 3.40 (± 3.75, 0–21) updates in the control group (Fig. 2, Table 2). Based on the negative binomial model, the incident rate in the intervention group was 1.34 times the incident rate of the control group (*p* = 0.044), holding the other variables (confounders) constant.

**Table 2:** Descriptive analysis and negative binomial model for the primary endpoint

	Intervention (N = 76)			Control (N = 75)			Overall (N = 151)		
	Mean (SD)	Range	N (%)	Mean (SD)	Range	N (%)	Mean (SD)	Range	N (%)
*Descriptive analysis*									
Updates per patient [N]	4.93 (± 4.45)	0–18	375 (59.5)	3.40 (± 3.75)	0–21	255 (40.5)	4.17 (± 4.18)	0–21	630 (100.0)

Parameter	Exp(B)	95%-Wald confidence interval for Exp(B)	Wald-χ^2^-test	*p* value					
*Negative binomial model*									
Constant term	0.62	0.29–1.31	1.57	0.210					
Intervention group (Reference: control group)	1.34	1.01–1.78	4.04	0.044*					

Dependent variable: Number (N) of updates per patient. Exp(B) = exponentiated estimate, N = number, SD = standard deviation. *p < 0.05

In our model, the *number of home medicines based on the PVML* and the *days elapsed since its documentation* showed a statistically significant effect on the number of updates (Supplement B). Subgroup analyses revealed a significant interaction between the *number of home medicines* (0–4, 5–9, ≥ 10 medicines) and the *group* (*p* < 0.001). There were larger effects in the subset “ ≥ 10” than in the other two (Supplement C). The significant interaction between *age* and *group* (*p* = 0.010) showed an increased likelihood of the numbers of updates with increasing age in both intervention (Exp(B) = 1.02, confidence interval (CI) = 1.00–1.03) and control (Exp(B) = 1.01, CI = 1.00–1.03) group. There were no significant interactions between *sex* and *group* (*p* = 0.241) or *days elapsed since documentation of PVML* (≤ 30, 31–90, ≥ 91 days) and *group* (*p* = 0.159). However, group effects per subset were larger in the subsets > 30 days compared to the subset ≤ 30.

### Key secondary endpoint

The occurrence of ≥ 1 update differed significantly between the groups. In contrast to the model of the primary endpoint, this model revealed a significant impact only for the number of home medicines based on the PVML, but not for the days elapsed since its documentation (Table 3).

**Table 3:** Logistic regression model for the binary endpoint “at least one update”

Parameter	Odds ratio	95%-Wald confidence interval for OR	Wald-χ^2^-test	*p* value
Constant term	0.21	–	1.53	0.217
Intervention group, Reference: control group	3.94	1.14–10.82	7.08	0.008*
Number of home medicines and medical devices based on PVML	1.29	1.10–1.51	9.97	0.002*
Age	1.01	0.98–1.04	0.47	0.492
Sex	0.45	0.17–1.16	2.74	0.098
log (days elapsed since documentation of the PVML)	1.69	0.72–3.97	1.43	0.233

Dependent variable: Number of updates per patient. Model: Constant term, group, number of home medicines & medical devices based on PVML, age, sex, log (time since documentation of the PVML). OR = Odds ratio, PVML = pre-visit medication list. *p < 0.05

### Exploratory secondary endpoints

In total, 548 updated medicines with 630 updates were found in all patients (Supplement D). The *number of updates in each category* was significantly higher in the category “changed medicines” in the intervention group (*p* < 0.001). The percentages of major ATC groups that were affected by updates were similar in the groups. The most commonly affected groups were: *Alimentary tract and metabolism* (42%) and *Cardiovascular system* (18%). Of 198 medicines with ≥ 1 change, the total number of changes was 280 with “dose” and “frequency” being the most common subtypes overall (45.5% and 32.5%,) and in the intervention (41.1% and 33.0%) and control group (53.7% and 31.6%). Detailed information on the subtypes is shown in Supplement E.

### Tertiary endpoints

In the intervention group medication history interview took a mean of 6.3 min (± 3.5; 1–23). The time required to document the medication history in the EMR was measured in 68/76 patients and resulted in a mean of 6.1 min (± 4.1; 1–19), while the switch to the in-house medication took 4.5 min (± 3.1; 0–13). The recording and documentation of the medication history took 15.7 min (± 9.8; 1–53). In the standard care group, the start and end time of documentation was extracted from the EMR in 29/75 patients and took 4.3 min (± 4.4, 1–18).

In total, 144 patients (95%) participated in the voluntary satisfaction survey (69 = intervention, 75 = control). Interviews by telephone were well received by the intervention group, and patients in the control group were generally receptive to this approach. The groups differed in preference for timing, discussing medication on the phone, and perceived usefulness for medical staff. An online platform was considered a potential alternative to provide medication information by 73.6% of the patients, whereas video-calls were less favoured (Table 4).

**Table 4:** Results of the patient survey

Question	Answer options	Intervention % (N/N_total_)	Control % (N/N_total_)	Total % (N/N_total_)	*p* value group comparison)
How important do you think it is to record medication at the start of your treatment in hospital?	Very important	76.1(51/67)	73.3 (55/75)	74.6 (106/142)	0.698
	Rather important	16.4 (11/67)	20.0 (15/75)	18.3 (26/142)	
	Rather unimportant	6.0 (4/67)	6.7 (5/75)	6.3 (9/142)	
	Unimportant	1.5 (1/67)	0.0 (0/75)	0.7 (1/142)	
How do you feel about the time it took to record your medicines by phone?	Too short	0.0 (0/69)	3.2 (2/63)	1.5 (2/129)	0.325
	Appropriate	95.7 (66/69)	92.1 (58/63)	93.9 (124/129)	
	Too long	4.3 (3/69)	4.8 (3/63)	4.5 (6/129)	
Medication are usually recorded during hospital admission. How did/would you feel about recording your medication the day before instead?	Better	73.9 (51/69)	34.7 (26/75)	53.5 (77/144)	< 0.001*
	The same	21.7 (15/69)	42.7 (32/75)	32.6 (47/144)	
	Worse	4.3 (3/69)	22.7 (17/75)	13.9 (20/144)	
Was it easier, the same or more difficult for you to list your medication at home as compared to listing it in hospital?	Easier	41.8 (28/67)			
	The same	52.2 (35/67)			
	More difficult	6.0 (4/67)			
How did you find the conversation about your medication over the phone compared to a face-to-face conversation?	Better	15.9 (11/69)			
	The same	75.4 (52/69)			
	Worse	8.7 (6/69)			
Would you be prepared to record your medication history by phone?	Yes		84.0 (63/75)		
	No		16.0 (12/75)		
Do you feel comfortable providing your medication history over the phone?	Yes	98.6 (68/69)	88.0 (66/75)	93.1 (134/144)	0.013*
	No	1.4 (1/69)	12.0 (9/75)	6.9 (10/144)	
Do you think it is or could be helpful for the staff (physicians and nurses) to record the medication before the day of admission?	Yes	93.7 (59/63)	70.4 (50/71)	81.3 (109/134)	< 0.001*
	No	6.3 (4/63)	29.6 (21/71)	18.7 (25/134)	
Would you be prepared to have your medication recorded in a video call?	Yes	50.7 (35/69)	34.7 (26/75)	42.4 (61/144)	0.051
	No	49.3 (34/69)	65.3 (49/75)	59.0 (83/144)	
Do you own a device (e.g. computer, smartphone, tablet) that could be used for a video call?	Yes	80.6 (54/67)	82.4 (61/74)	81.6 (115/141)	0.779
	No	19.4 (13/67)	17.6 (13/74)	18.4 (26/141)	
Would you enter your medication information on a hospital platform (e. g. online patient portal) before the day of admission?	Yes	79.7 (55/69)	68.0 (51/75)	73.6 (106/144)	0.111
	No	20.3 (14/69)	32.0 (24/75)	26.4 (38/144)	

Group comparisons were conducted using the Pearson-χ^2^-test. *p < 0.05

Staff satisfaction was evaluated two weeks after start of the intervention (survey 1) and three weeks after the end (survey 2) with the same N = 4 participating physicians. The estimated mean daily time savings were 25 min (10–40) in survey 1 and 7.5 min (0–10) in survey 2. Satisfaction with the medication history quality decreased from 100% full satisfaction to 50% full and 50% partial satisfaction (Supplement F).

## Discussion

This project aimed to test a new study design and quality parameter to evaluate medication history taking: It assesses updates instead of discrepancies to avoid taking two medication histories. We used this design to evaluate the feasibility of medication history taking by telephone. The design and the results of the feasibility study will be discussed separately.

### Methodology

Our design aimed to provide a low-resource alternative to evaluate the quality of medication histories. The gold standard requires additional, time-intensive interviews (on average 30 min [4]) and places burden on patients. Our quality parameter requires only one interview per patient, as existing documents (PVML) serve as comparators instead of additionally acquired BPMHs. This reduces the time spent on patients and research resources.

Beforehand, there were two expected weaknesses of our design [15]: First, the availability of the PVML was expected to limit patient recruitment, but only 10% of patients lacked PVML information. The most common exclusion reason was discharge before written consent could be obtained. This might be linked to our specific intervention being conducted remotely and requiring an additional in-person contact, which is consistent with a similar study [25], but would not apply to all interventions. At this point, our design is suitable for settings with recurring admissions. With increasing standardization in documentation and the introduction of broad consent, these weaknesses will not affect the feasibility of the design significantly.

This pilot study revealed an important consideration in regard to the PVML: Although the distribution of days elapsed since its documentation in the patient population was relatively symmetric, we encountered a few outliers and a wide range (3 days to 15 years). Our exploratory analyses indicated a potentially significant impact of this factor on the primary endpoint. Therefore, future studies should introduce a maximum number of days elapsed as exclusion criterion or conduct stratified analyses. A previous study showed that most medication lists > 30 days old are outdated [28]. However, the appropriate maximum may also depend on the frequency of hospital visits in the studied population. In our setting, a maximum of 90 days, the usual time period between two visits, could have been set.

Secondly, our outcome parameter assumes that all updates are changes made to a patient’s medication therapy in primary care and, therefore, are correct, but naturally they could also be caused by errors in history taking. Like the BPMH technique, we followed a systematic approach for the interviews with specific questions to cover all types of medicines. Further, we used the PVML to verify apparent changes. Most updates found in our study were “changed” and “newly initiated” medicines, which we consider expectable modifications in the course of therapy. This was confirmed by the similar distribution of types of updates in both groups. In previous research, the most common discrepancies were omissions [29, 30], which indicates that dose and commission errors are less likely. Our BPMH phase showed that most discrepancies between standard-care histories and BPMH were caused by the PVML being adopted unchanged, not by recording incorrect information. We therefore believe that our outcome parameter—despite higher uncertainty compared to the gold standard—is a sufficiently reliable proxy. However, it has to be validated by comparison with the BPMH method.

### Medication history taking by telephone

Telephone interviews proved feasible with a mean time expenditure of 15.7 min per patient. In general, patients were open to medication history taking by telephone in both groups, which aligns with previous studies [31]. The exploratory staff surveys showed that physicians were satisfied with the intervention and suggests 0–40 min saved daily per physician. Recording medication histories before admission could distort the admission day and render the telephone approach especially useful in time-sensitive settings with high workload and patient turnover.

The statistically significant increase in the number of updates in favour of the intervention group suggests that the telephone approach might result in more comprehensive medication histories. However, we did not formally evaluate the clinical significance of this difference. Our analyses showed that updates mostly affected medicines used in the alimentary system (mainly anti-diabetics, drugs for acid-related disorders) and cardiovascular system (mainly diuretics, beta-blockers, lipid modifying agents), particularly update types “newly initiated” and “changed”. A previous study found the same drug classes to be affected the most by medication discrepancies (predominantly omissions and discrepancies in dose and/or frequency). Additionally, this study reported that errors concerning cardiovascular medicines and antidiabetics were potentially harmful [32]. Therefore, we assume that *not* identifying updates in these medicines might accordingly increase the risk of harm.

Similar to previous studies, we identified factors associated with a higher number of updates: hyperpolypharmacy (≥ 10 home medicines), increasing age, and a PVML documented ≥ 30 days before admission. Although these associations remain inconclusive across studies [28, 33–36], patients with these characteristics should be assessed more closely.

The observed difference in the number of updates could be explained by several aspects in which our intervention differed from standard-care: Histories were taken by a pharmacist instead of medical staff. It has been shown that these professions differ in the type of medicines they record [37] and that pharmacists tend to gather more comprehensive histories [31, 38–40]. Additionally, the pharmacist used a standardised form in our study, while physicians and medical interns used individual forms. Standardisation may positively impact quality according to literature [31, 41–43]. Although this constitutes a source of bias in favour of the intervention, we tried to minimize it by only including medicines and medicinal products in the analyses and by providing the pharmacist with the same information sources as the physicians (i. e., patient documents in the EMR). We believe that the earlier timing of the telephone interviews is the most important factor due to potentially fewer interruptions, which are generally common in admission interviews [4]. A previous study associated this with high completion rates of medication histories, because patients had easier access to their medicines at home [22], which corresponds with the results of our patient survey: Approximately 42% of patients found it easier to list their medicines from home. In other studies, the telephone approach provided accurate medication histories and was faster than face-to-face interviews with values between 5.8 and 8.25 min [23, 25], which is comparable to our results (6.3 min). However, to provide robust evidence on the individual impact of the aforementioned aspects, they would need to be studied separately or in a factorial study design. This was infeasible in our setting.

Based on the time measured, the staff survey and EMR data, pharmacist-acquired histories by telephone were more time-consuming than in-person interviews by physicians. Conversely, according to our primary endpoint, the telephone interviews were of better quality. This phenomenon is known as the *efficiency-thoroughness-trade-off* [44] and suggests that better quality might require (and potentially justify) a more time. However, due to the limited comparability regarding time expenditure (see *Limitations*) and the uncertain clinical relevance of the difference between the groups, a formal cost-effectiveness analysis was not undertaken. Apart from potential time savings and reduced admission-day workload for physicians, telephone histories might streamline subsequent processes (e.g., medication orders) and could, thereby, positively impact cost-effectiveness.

While the telephone approach was well accepted by patients, more than half of the survey participants rejected medication history taking via video-call, despite > 80% owning a suitable electronic device. Although our survey questionnaires do not enable qualitative content analysis due to the close-ended question format, individual comments of the participants suggested that video-calls may be considered too cumbersome. Online platforms were favoured, indicating a general receptiveness for digital formats in this population. However, for their implementation, factors such as age, digital and health literacy and general trust need to be considered [19].

### Limitations

The major limitation of this study is the uncertainty of our quality parameter. The BPMH phase was performed on small sample size of six patients and cannot be considered a full validation. Accordingly, all *p*-values must be interpreted exploratively. Next, our quality parameter will be validated against the BPMH or medication discrepancies.

About 90% of patients assigned to the intervention completed the telephone histories. Even though telephone interviews were feasible for them, we managed to obtain written consent from only 56%. Patients not answering the phone were excluded, which could have biased the study, as telephone calls may be infeasible or undesirable for them.

We relied on estimates and data from the EMR for the time spent on medication histories in the control group. This might introduce bias, as interruptions cannot be accounted for, but reflects the reality of clinical practice. Physicians should have been monitored closely to obtain reliable data on time expenditure, however, this would have introduced observer bias.

Lastly, the questionnaires were not formally validated. Additionally, only four physicians participated in the survey on staff satisfaction. This small sample size reflected the staffing levels at the study sites with only two physicians on duty per ward. Due to high workload, the willingness to participate in this non-mandatory survey was low. Naturally, the results of the staff survey are exploratory and the low response rate limits their generalizability.

## Conclusion

We were able to evaluate medication history taking by telephone with our new design. Before application in future studies, it should be validated against the gold standard to test its reliability. The results suggest that the quality of medication histories taken by telephone were slightly better than those acquired by face-to-face interviews. The telephone approach was well received by patients and could potentially streamline patient admission. Also, online platforms were favoured by patients and should be explored further.

## Supplementary Information

Below is the link to the electronic supplementary material.

Supplementary file1 (PDF 225 KB)

Supplementary file2 (PDF 155 KB)

Supplementary file3 (PDF 182 KB)

Supplementary file4 (PDF 152 KB)

Supplementary file5 (PDF 212 KB)

Supplementary file6 (PDF 156 KB)

## Data Availability

The datasets generated during and/or analysed during the current study are available from the corresponding author on reasonable request.

## References

[1] Fitzgerald RJ. Medication errors: the importance of an accurate drug history. Br J Clin Pharmacol. 2009;67(6):671–5. 10.1111/j.1365-2125.2009.03424.x. 19594536 10.1111/j.1365-2125.2009.03424.x PMC2723207

[2] Silvestre CC, Santos LMC, de Oliveira-Filho AD, et al. ‘What is not written does not exist’: the importance of proper documentation of medication use history. Int J Clin Pharm. 2017;39(5):985–8. 10.1007/s11096-017-0519-2. 28823070 10.1007/s11096-017-0519-2

[3] Rodríguez Vargas B, Delgado Silveira E, Iglesias Peinado I, et al. Prevalence and risk factors for medication reconciliation errors during hospital admission in elderly patients. Int J Clin Pharm. 2016;38(5):1164–71. 10.1007/s11096-016-0348-8. 27558355 10.1007/s11096-016-0348-8

[4] Ghazanfar MN, Honoré PH, Nielsen TRH, et al. Hospital admission interviews are time-consuming with several interruptions. Dan Med J. 2012;59(12):A4534. 23290281

[5] Rivera-Rodriguez AJ, Karsh B-T. Interruptions and distractions in healthcare: review and reappraisal. Qual Saf Health Care. 2010;19(4):304–12. 10.1136/qshc.2009.033282. 20378621 10.1136/qshc.2009.033282 PMC3007093

[6] Laxmisan A, Hakimzada F, Sayan OR, et al. The multitasking clinician: decision-making and cognitive demand during and after team handoffs in emergency care. Int J Med Inform. 2007;76(11–12):801–11. 10.1016/j.ijmedinf.2006.09.019. 17059892 10.1016/j.ijmedinf.2006.09.019

[7] Henriksen JP, Noerregaard S, Buck TC, et al. Medication histories by pharmacy technicians and physicians in an emergency department. Int J Clin Pharm. 2015;37(6):1121–7. 10.1007/s11096-015-0172-6. 26243529 10.1007/s11096-015-0172-6

[8] Lea M, Barstad I, Mathiesen L, et al. Effect of teaching and checklist implementation on accuracy of medication history recording at hospital admission. Int J Clin Pharm. 2016;38(1):20–4. 10.1007/s11096-015-0218-9. 26589204 10.1007/s11096-015-0218-9

[9] Huber T, Brinkmann F, Lim S, et al. Implementation of an IT-guided checklist to improve the quality of medication history records at hospital admission. Int J Clin Pharm. 2017;39(6):1312–9. 10.1007/s11096-017-0545-0. 29082460 10.1007/s11096-017-0545-0 PMC5694519

[10] Francis M, Deep L, Schneider CR, et al. Accuracy of best possible medication histories by pharmacy students: an observational study. Int J Clin Pharm 2022;45(2):414–420. 10.1007/s11096-022-01516-2 10.1007/s11096-022-01516-2 PMC9749631 36515780

[11] Mazhar F, Haider N, Ahmed Al-Osaimi Y, et al. Prevention of medication errors at hospital admission: a single-centre experience in elderly admitted to internal medicine. Int J Clin Pharm. 2018;40(6):1601–13. 10.1007/s11096-018-0737-2. 30367379 10.1007/s11096-018-0737-2

[12] Abdulghani KH, Aseeri MA, Mahmoud A, et al. The impact of pharmacist-led medication reconciliation during admission at tertiary care hospital. Int J Clin Pharm. 2018;40(1):196–201. 10.1007/s11096-017-0568-6. 29248986 10.1007/s11096-017-0568-6

[13] Kabir R, Liaw S, Cerise J, et al. Obtaining the best possible medication history at hospital admission: description of a pharmacy technician-driven program to identify medication discrepancies. J Pharm Pract. 2023;36(1):19–26. 10.1177/08971900211021254. 34080461 10.1177/08971900211021254

[14] Meguerditchian AN, Krotneva S, Reidel K, et al. Medication reconciliation at admission and discharge: a time and motion study. BMC Health Serv Res. 2013;13:485. 10.1186/1472-6963-13-485. 24261516 10.1186/1472-6963-13-485 PMC3842651

[15] Terstegen T, Kirchner M, Haefeli WE, et al. Proposal for a new study design and endpoint in research on medication history taking. J Pharm Policy Pract. 2024;17(1):2396967. 10.1080/20523211.2024.2396967. 39253622 10.1080/20523211.2024.2396967 PMC11382705

[16] Yap M, Edwards G, Gibbs H, et al. A cohort study comparing pharmacist activities during participation in general medical ward rounds: telehealth versus in-person during the COVID-19 pandemic. Int J Clin Pharm. 2024;46(2):522–8. 10.1007/s11096-024-01701-5. 38368283 10.1007/s11096-024-01701-5

[17] Baldoni S, Amenta F, Ricci G. Telepharmacy services: present status and future perspectives: a review. Medicina (Kaunas). 2019;55(7):327. 10.3390/medicina55070327. 31266263 10.3390/medicina55070327 PMC6681067

[18] Hursman A, Vang C, Thooft T, et al. The role of telepharmacy in the delivery of clinical pharmacy services following the Covid-19 pandemic: a descriptive report. J Pharm Technol. 2024;40(2):66–71. 10.1177/87551225231222426. 38525089 10.1177/87551225231222426 PMC10959079

[19] Chong RLK, Chan ASE, Chua CMS, et al. Telehealth interventions in pharmacy practice: systematic review of reviews and recommendations. J Med Internet Res. 2025;27:e57129. 10.2196/57129 10.2196/57129 PMC12096025 40334268

[20] Marchese M, Heintzman A, Pasetka M, et al. Development of a process map for the delivery of virtual clinical pharmacy services at Odette Cancer Centre during the COVID-19 pandemic. J Oncol Pharm Pract. 2021;27(3):650–7. 10.1177/1078155221991202. 33554738 10.1177/1078155221991202 PMC8008431

[21] McGinnis B, Padilla E, Garret P, et al. Using pharmacy technicians and telepharmacy to obtain medication histories in the emergency department. J Am Pharm Assoc. 2019;59(3):390–397. 10.1016/j.japh.2019.01.019 10.1016/j.japh.2019.01.019 30853346

[22] Bormann TM, Brower KI, Forshay CM. Implementation of pharmacy-led preoperative medication reconciliation in surgical oncology patients. J Am Pharm Assoc. 2024;64(2):582–587. 10.1016/j.japh.2024.01.006 10.1016/j.japh.2024.01.006 38218584

[23] Francis M, Francis P, Patanwala AE, et al. Obtaining medication histories via telepharmacy: an observational study [published correction appears in J Pharm Policy Pract. 2023;16(1):80. 10.1186/s40545-023-00588-3]. J Pharm Policy Pract. 2023;16(1):69. 10.1186/s40545-023-00573-w. 10.1186/s40545-023-00573-w PMC10248974 37291672

[24] Gadallah A, McGinnis B, Nguyen B, et al. Assessing the impact of virtual medication history technicians on medication reconciliation discrepancies. Int J Clin Pharm. 2021;43(5):1404–11. 10.1007/s11096-021-01267-6. 33871769 10.1007/s11096-021-01267-6

[25] Canning ML, Vale C, Wilczynski H, et al. Comparison of medication history interview conducted via telephone with interview conducted face-to-face for elective surgical patients. J Pharm Pract Res. 2018;48(4):334–9. 10.1002/jppr.1402.

[26] Terstegen T, Bittmann JA, Jungreithmayr V, et al. Evaluation Der Telefonischen Arzneimittelanamnese Bei Elektiven Patienten Auf Einer Gastroenterologischen Station [poster abstract]. Krankenhauspharmazie. 2023;44(01):38.

[27] Terstegen T, Jungreithmayr V, Bittmann J, et al. Patients’ perception and acceptance of medication history taking via telephone [poster abstract]. Pharm Educ. 2023;23(5):26–7.

[28] Amelung S, Bender B, Meid A, et al. Wie vollständig ist der Bundeseinheitliche Medikationsplan? Eine Analyse bei Krankenhausaufnahme [How complete is the Germany-wide standardised medication list ("Bundeseinheitlicher Medikationsplan")? An analysis at hospital admission.] Dtsch Med Wochenschr. 2020;145(21):e116–e122. 10.1055/a-1212-2836 10.1055/a-1212-2836 PMC7575356 33022741

[29] Cornish PL, Knowles SR, Marchesano R, et al. Unintended medication discrepancies at the time of hospital admission. Arch Intern Med. 2005;165(4):424–9. 10.1001/archinte.165.4.424. 15738372 10.1001/archinte.165.4.424

[30] Tam VC, Knowles SR, Cornish PL, et al. Frequency, type and clinical importance of medication history errors at admission to hospital: a systematic review. CMAJ. 2005;173(5):510–5. 10.1503/cmaj.045311. 16129874 10.1503/cmaj.045311 PMC1188190

[31] Terstegen T, Niestroj C, Stangl J, et al. Approaches to medication history taking in different hospital settings: a scoping review. Am J Health Syst Pharm. 2024;81(15):e419–30. 10.1093/ajhp/zxae112. 38660785 10.1093/ajhp/zxae112

[32] Gleason K, McDaniel MR, Feinglass J, et al. Results of the medications at transitions and clinical handoffs (match) study: an analysis of medication reconciliation errors and risk factors at hospital admission. J Gen Intern Med. 2010;25(5):441–7. 10.1007/s11606-010-1256-6. 20180158 10.1007/s11606-010-1256-6 PMC2855002

[33] van der Luit CD, de Jong IR, Ebbens MM, et al. Frequency of occurrence of medication discrepancies and associated risk factors in cases of acute hospital admission. Pharm Pract (Granada). 2018;16(4):1301. 10.18549/PharmPract.2018.04.1301. 30637032 10.18549/PharmPract.2018.04.1301 PMC6322986

[34] Abu Farha R, Yousef A, Gharaibeh L, et al. Medication discrepancies among hospitalized patients with hypertension: assessment of prevalence and risk factors. BMC Health Serv Res. 2021;21(1):1338. 10.1186/s12913-021-07349-5. 34903221 10.1186/s12913-021-07349-5 PMC8670213

[35] Giannini O, Rizza N, Pironi M, et al. Prevalence, clinical relevance and predictive factors of medication discrepancies revealed by medication reconciliation at hospital admission: prospective study in a Swiss internal medicine ward. BMJ Open. 2019;9(5): e026259. 10.1136/bmjopen-2018-026259. 31133583 10.1136/bmjopen-2018-026259 PMC6538074

[36] Moges TA, Akalu TY, Sema FD. Unintended medication discrepancies and associated factors upon patient admission to the internal medicine wards: identified through medication reconciliation. BMC Health Serv Res. 2022;22(1):1251. 10.1186/s12913-022-08628-5. 36243696 10.1186/s12913-022-08628-5 PMC9571466

[37] Carow F, Rieger K, Walter-Sack I, et al. Objective assessment of nonadherence and unknown co-medication in hospitalized patients. Eur J Clin Pharmacol. 2012;68(8):1191–9. 10.1007/s00228-012-1229-2. 22354152 10.1007/s00228-012-1229-2

[38] Arrison W, Merritt E, Powell A. Comparing medication histories obtained by pharmacy technicians and nursing staff in the emergency department. Res Soc Adm Pharm. 2020;16(10):1398–400. 10.1016/j.sapharm.2020.01.009. 10.1016/j.sapharm.2020.01.009 32001155

[39] Lau HS, Florax C, Porsius AJ, et al. The completeness of medication histories in hospital medical records of patients admitted to general internal medicine wards. Br J Clin Pharmacol. 2000;49(6):597–603. 10.1046/j.1365-2125.2000.00204.x. 10848724 10.1046/j.1365-2125.2000.00204.x PMC2015045

[40] Akwagyriam I, Goodyer LI, Harding L, et al. Drug history taking and the identification of drug related problems in an accident and emergency department. J Accid Emerg Med. 1996;13(3):166–8. 10.1136/emj.13.3.166. 10.1136/emj.13.3.166 PMC1342679 8733649

[41] Chan AHY, Garratt E, Lawrence B, et al. Effect of education on the recording of medicines on admission to hospital. J Gen Intern Med. 2010;25(6):537–42. 10.1007/s11606-010-1317-x. 10.1007/s11606-010-1317-x PMC2869408 20237959

[42] McDonald D, Mansukhani R, Kokotajlo S, et al. Effect of nursing education on optimization of medication reconciliation in the pediatric emergency department. J Pediatr Pharmacol Ther. 2018;23(3):203–8. 10.5863/1551-6776-23.3.203. 29970976 10.5863/1551-6776-23.3.203 PMC6027975

[43] Wang T, Biederman S. Enhance the accuracy of medication histories for the elderly by using an electronic medication checklist. Perspect Health Inf Manag. 2012;9(Fall):1–15. 23209450 PMC3510644

[44] Hollnagel E. The ETTO principle: efficiency-thoroughness trade-off. CRC Press. 2017. 10.1201/9781315616247.

