# Supplementary material for: Evaluating the quality, feasibility and patient satisfaction of medication history taking by telephone for patients with scheduled admissions: a pilot study
Source: Int J Clin Pharm. 2025 Sep 8;48(2):479–89. doi: 10.1007/s11096-025-02002-1 (PMC12992431; doi:10.1007/s11096-025-02002-1)
Supplement: Supplementary file 2 — Supplementary file2 (PDF 155 KB) [file 11096_2025_2002_MOESM2_ESM.pdf]

Evaluating the quality, feasibility and patient satisfaction of medication history taking by telephone for patients with planned admissions to two gastroenterology wards

– Supplement B –

Theresa Terstegen[a], Janina Bittmann[a], Luise Kauk[a], Marietta Kirchner[b], Sebastian Krug[c], Annika Gauss[c], Ute Chiriac[d], Benedict Morath[d], Walter E. Haefeli[a], Hanna M. Seidling[a]

[a]Heidelberg University, Medical Faculty Heidelberg / Heidelberg University Hospital, Internal Medicine IX, Clinical Pharmacology and Pharmacoepidemiology, Cooperation Unit Clinical Pharmacy, Im Neuenheimer Feld 410, 69120 Heidelberg, Germany.

[b]Heidelberg University, Medical Faculty Heidelberg / Heidelberg University Hospital, Institute of Medical Biometry, Im Neuenheimer Feld 103.3, 69120 Heidelberg, Germany.

[c]Heidelberg University, Medical Faculty Heidelberg / Heidelberg University Hospital, Internal Medicine IV, Department of Gastroenterology, Infectiology and Toxicology, Im Neuenheimer Feld 410, 69120 Heidelberg, Germany.

[d]Heidelberg University, Medical Faculty Heidelberg / Heidelberg University Hospital, Hospital Pharmacy, Im Neuenheimer Feld 670, 69120 Heidelberg, Germany.

International Journal of Clinical Pharmacy

Corresponding Author

Prof. Dr. sc. hum. Hanna M. Seidling

Heidelberg University, Medical Faculty Heidelberg / Heidelberg University Hospital, Internal Medicine IX, Clinical Pharmacology and Pharmacoepidemiology, Cooperation Unit Clinical Pharmacy, Im Neuenheimer Feld 410, 69120, Heidelberg, Germany. hanna.seidling@med.uni-heidelberg.de

Supplement B: Negative binomial model for interaction effects on the primary endpoint, i. e., the number of updates per patient.

**Parameter**	**Exp(B)**	**95%-Wald-CI for Exp(B)**	**Wald-χ^2-test**	**p value**
Number of home medicines & medical devices based on PVML	1.11	1.07–1.15	29.73	**< 0.001**
Age	1.01	1.00–1.02	0.96	0.327
Sex	1.00	0.74–1.34	0.00	0.989
log (days elapsed since documentation of the PVML)	1.43	1.14–1.78	9.69	**0.002**

Dependent variable: Number of updates per patient. Model: Constant term, group, number of home medicines & medical devices based on PVML, age, sex, log (days elapsed since documentation of the PVML). CI = confidence interval, Exp(B) = exponentiated estimate, PVML = Pre-visit medication list.
