# Supplementary material for: Evaluating the quality, feasibility and patient satisfaction of medication history taking by telephone for patients with scheduled admissions: a pilot study
Source: Int J Clin Pharm. 2025 Sep 8;48(2):479–89. doi: 10.1007/s11096-025-02002-1 (PMC12992431; doi:10.1007/s11096-025-02002-1)
Supplement: Supplementary file 6 — Supplementary file6 (PDF 156 KB) [file 11096_2025_2002_MOESM6_ESM.pdf]

Evaluating the quality, feasibility and patient satisfaction of medication history taking by telephone for patients with planned admissions to two gastroenterology wards

– Supplement F –

Theresa Terstegen[a], Janina Bittmann[a], Luise Kauk[a], Marietta Kirchner[b], Sebastian Krug[c], Annika Gauss[c], Ute Chiriac[d], Benedict Morath[d], Walter E. Haefeli[a], Hanna M. Seidling[a]

[a]Heidelberg University, Medical Faculty Heidelberg / Heidelberg University Hospital, Internal Medicine IX, Clinical Pharmacology and Pharmacoepidemiology, Cooperation Unit Clinical Pharmacy, Im Neuenheimer Feld 410, 69120 Heidelberg, Germany.

[b]Heidelberg University, Medical Faculty Heidelberg / Heidelberg University Hospital, Institute of Medical Biometry, Im Neuenheimer Feld 103.3, 69120 Heidelberg, Germany.

[c]Heidelberg University, Medical Faculty Heidelberg / Heidelberg University Hospital, Internal Medicine IV, Department of Gastroenterology, Infectiology and Toxicology, Im Neuenheimer Feld 410, 69120 Heidelberg, Germany.

[d]Heidelberg University, Medical Faculty Heidelberg / Heidelberg University Hospital, Hospital Pharmacy, Im Neuenheimer Feld 670, 69120 Heidelberg, Germany.

International Journal of Clinical Pharmacy

Corresponding Author

Prof. Dr. sc. hum. Hanna M. Seidling

Heidelberg University, Medical Faculty Heidelberg / Heidelberg University Hospital, Internal Medicine IX, Clinical Pharmacology and Pharmacoepidemiology, Cooperation Unit Clinical Pharmacy, Im Neuenheimer Feld 410, 69120, Heidelberg, Germany. hanna.seidling@med.uni-heidelberg.de

Supplement F. Results of the staff satisfaction survey on medication history taking by telephone of N = 4 physicians.

Question	Answer options	Survey 1 (N = 4)	Survey 2 (N = 4)
Has the new concept decreased time expenditure for medication history taking?	Yes	100 %	75 %
	No, unchanged	0 %	25 %
	No, additional expenditure	0 %	0 %
Were you satisfied with the quality of the medication histories taken?	Yes	100 %	50 %
	Partly	0 %	50 %
	No	0 %	0 %
Has the quality of the medication history changed as a result of the new concept compared to the routine care?	Yes, improved	75 %	25 %
	No, unchanged	25 %	75 %
	Yes, worsened	0 %	0 %
Has drug therapy safety improved as a result of the new concept compared to the routine care?	Yes, improved	75 %	25 %
	No, unchanged	25 %	75 %
	Yes, worsened	0 %	0 %
Would you like the new concept to become routine care?	Yes	75 %	100 %
	No	25 %	0 %
What other components would you like to see in this project?	Information on drug-related problems	50 %	50 %
	Information on drug interactions	75 %	50 %
	Information on dose adjustments for impaired kidney function	75 %	75 %
	Recommendations for admission medication orders (in-house)	100 %	100 %
	Other	0 %	0 %
